# Association of serum inflammasome proteins and pediatric traumatic brain injury severity

**DOI:** 10.1038/s41390-025-04410-5

**Published:** 2025-09-26

**Authors:** Jennifer C. Munoz Pareja, Maria B. Mateo Chavez, Julia Alexis Bernal, Kathryn Swaby, Natalie Machado, Charlene Pringle, Kourtney Guthrie, Jennifer Coto, Dhanashree Rajderkar, Joslyn Gober, Juan Solano, Heather J. McCrea, Daniel Gonzalez Mosquera, Ayham Alkhachroum, Kristine H. O’Phelan, Firas Kobeissy, Robert W. Keane, Kevin K. Wang, W. Dalton Dietrich, Juan Pablo de Rivero Vaccari

**Affiliations:** 1 Department of Pediatric Critical Care, University of Miami Miller School of Medicine, Miami, FL USA; 2 The Miami Project to Cure Paralysis, University of Miami Miller School of Medicine, Miami, FL USA; 3 Knowledge and Evaluation Research Unit, Mayo Clinic, Rochester, MN USA; 4 University of Miami Miller School of Medicine, Miami, FL USA; 5 Department of Pediatrics, Critical Care, University of Florida College of Medicine, Gainesville, FL USA; 6 University of Miami Concussion Program, Department of Otolaryngology, University of Miami Miller School of Medicine, Miami, FL USA; 7 Department of Radiology, Division of Pediatric Radiology, University of Florida College of Medicine, Gainesville, FL USA; 8 Department of Pediatric Rehabilitation, University of Miami Miller School of Medicine, Miami, FL USA; 9 Department of Neurological Surgery and Pediatrics, University of Miami Miller School of Medicine/Jackson Health System, Miami, FL USA; 10 Department of Internal Medicine, Lincoln Medical Center, Bronx, NY USA; 11 Neurocritical Care, Department of Neurology, University of Miami Miller School of Medicine, Miami, FL USA; 12 Department of Neurobiology, Morehouse University, School of Medicine, Atlanta, GA USA; 13 Center for Neurotrauma, Multiomics & Biomarkers (CNMB), Morehouse University, School of Medicine, Atlanta, GA USA; 14 Department of Neurological Surgery and the Miami Project to Cure Paralysis, University of Miami Miller School of Medicine, Miami, FL USA; 15 Department of Physiology and Biophysics, University of Miami Miller School of Medicine, Miami, FL USA

## Abstract

**Background:**

Pediatric traumatic brain injury (pTBI) often leads to cognitive, behavioral, and motor impairments. NLRP3 inflammasome proteins, such as ASC and caspase-1, may serve as biomarkers for TBI severity due to their role in neuroinflammation. This study aims to assess the association between serum ASC and caspase-1 levels and TBI severity in pediatric patients.

**Methods:**

Serum samples were collected at pediatric intensive care unit (ICU) admission (first post-admission), and at 24 and 48 h post-admission, from TBI participants aged 28 days to 18 years and from demographically matched controls. TBI severity was assessed using the Glasgow Coma Scale (GCS).

**Results:**

We analyzed samples from 77 pTBI patients and 31 controls. ASC levels were significantly higher across all GCS categories, with the most pronounced differences in the severe category at first post-admission (*p* = 0.0005, AUROC 0.83) and 24 h post-admission (*p* < 0.0001, AUROC 0.83). Caspase-1 levels were significantly elevated in the severe category, particularly at first post-admission (*p* < 0.0001, AUROC 0.85).

**Discussion:**

Elevated ASC and caspase-1 levels, especially in severe pTBI cases, suggest their potential as biomarkers for TBI severity. These findings emphasize the role of inflammasome proteins in post-TBI neuroinflammation and support further research into targeted therapies for pediatric TBI.

**Impact:**

- Increased serum levels of inflammasome proteins ASC and caspase-1 in acute-phase post-admission samples are associated with severe TBI.
- To our knowledge, this is the first study to examine the inflammasome pathway in pediatric TBI patients across the severity spectrum using serum samples.
- The study enhances our understanding of NLRP3 inflammasome activation in pediatric TBI by profiling serum levels and examining their clinical correlation with injury severity.
- It suggests an adjunctive approach to the Glasgow Coma Scale with biomarkers for more precise TBI diagnosis.
- This research lays the groundwork for future therapeutic strategies targeting inflammasomes in pediatric TBI.

## Introduction

Pediatric traumatic brain injury (pTBI) is a significant cause of pediatric morbidity and mortality, leading to approximately 475,000 annual emergency room visits among children aged 0–14 years.^1^ Long-term consequences of pTBI can result in enduring impairments in behavioral, cognitive, motor, and vocational functions, making it a critical public health concern.^2,3^ Its pathophysiology involves primary and secondary injuries. The primary injury is immediate brain damage caused by mechanical force, while the secondary injury encompasses a cascade of neurochemical and metabolic events that exacerbate brain damage, potentially persisting for years.^4^

A central factor in secondary injury is the neuroinflammatory response, in which the inflammasome pathway plays a crucial role. Inflammasomes are multiprotein complexes that trigger the innate immune response by detecting damage-associated molecular patterns released from injured tissues.^5^ Subsequently, these events lead to the upregulation of pro-inflammatory mediators such as tumor necrosis factor, interleukin (IL) 6, and IL-1β, which are early drivers of post-traumatic neuroinflammation.^6^ The NLRP3 inflammasome is the most studied and characterized inflammasome due to its ability to be activated by a diverse array of stimuli, prompting its cytosolic assembly and subsequent release into the extracellular fluid and circulation, with implications in several inflammatory diseases such as Alzheimer’s disease, diabetes, and atherosclerosis.^7–10^

Preclinical studies have demonstrated NLRP3 activation’s deleterious effects in hypoxic-ischemic brain injury in newborn animal models.^11^ In clinical traumatic brain injury (TBI) research, NLRP3 inflammasome-associated proteins have been evaluated as potential injury biomarkers in cerebrospinal fluid (CSF) and serum samples of adult patients, with results showing increased levels of NLRP3 related proteins such as caspase-1 increased in concordance with clinical markers of injury severity, such as elevated intracranial pressure, as well as with poor neurologic outcomes.^12^ In pediatric patients, however, these proteins have only been studied in CSF by a single-center study, where higher levels of NLRP3 proteins were found in pTBI patients’ samples compared to controls across all time points.^13^ Nevertheless, a gap in current clinical knowledge exists regarding post-TBI inflammasome serum profiles and their association with pTBI severity. Exploring the temporal relationship between these proteins and TBI may provide insights into the role of innate immunity in early pTBI pathophysiology, potentially aiding in strategies to modulate the resulting inflammatory cascade, with possible implications in neurologic outcomes.

This study aimed to evaluate the association of serum levels of NLRP3 inflammasome, specifically apoptosis-associated speck-like protein containing a caspase recruitment domain (ASC) and caspase-1, as candidate biomarkers of neuroinflammatory activation following pediatric TBI. These inflammasome-related proteins may reflect early innate immune activation and serve both as potential indicators of injury severity and as exploratory mechanistic markers of NLRP3 pathway involvement. We hypothesized that serum levels of these proteins would be elevated correspondingly to pTBI severity in participants versus control subjects.

## Methods

### Study design and setting

This prospective observational cohort study was conducted in the pediatric intensive care units (ICUs) of two academic medical centers in Florida, USA, from February 2017 to June 2023. This study adhered to the Strengthening the Reporting of Observational Studies in Epidemiology reporting guideline for cohort studies.^14^

### Participants

We recruited eligible participants aged 28 days to 18 years who were admitted or transferred to the pediatric ICUs of the medical centers. In accordance with our Institutional Review Board (IRB) approved protocol for the University of Florida (IRB201600237) and the University of Miami (IRB 20210352), we obtained written informed consent from a parent or guardian for participants under 18 years, while participants who were 18 years old provided consent on their own behalf. We approached eligible participants or their legal representatives immediately upon admission at the University of Miami and within 72 h after admission at the University of Florida by trained research staff. Recruitment was conducted in a manner to minimize selection bias, ensuring that all eligible patients during the study period were screened and approached systematically based on their admission time and clinical status. Inclusion criteria were a clinical diagnosis of TBI upon arrival. We defined TBI as a brain injury caused by an outside force.^15^ We excluded participants with psychiatric disorders exhibiting severe features as per the Diagnostic and Statistical Manual of Mental Disorders (Fifth Edition), pregnancy, or insufficient data for statistical analysis.^16^ Patients with known systemic infections or diagnosed autoimmune conditions were also excluded. Controls were selected if they fulfilled similar demographic characteristics as the selected participants, and were recruited from the pediatric emergency department, pediatric inpatient floor, and outpatient sedation services from both medical centers. Exclusion criteria for controls included a history of pTBI, pregnancy, acute neurologic dysfunction, or psychiatric disorders exhibiting severe features.^17^ We assessed TBI severity using a modified pediatric version of the Glasgow Coma Scale (GCS) for participants aged 2 years or younger or who were pre-verbal, and we used the standard GCS upon admission for both participants and controls.^18,19^ We used the lowest GCS score recorded for each participant for the final analysis.

### Data collection

We collected TBI-Common Data Elements (TBI-CDE), caregiver-reported demographic information, and detailed clinical assessments, as well as a seven-day compilation of relevant critical care data points extracted from electronic health records (EHR).^20^ All drugs administered at ICU admission were abstracted from the EHR. Medication classes specifically tracked included antiseizure, anti-inflammatory (NSAID or corticosteroid), and sedative/anxiolytic agents.

We followed the TBI-CDE Biospecimens and Biomarkers Working Group Consensus guidelines for serum preparation.^21^ Blood samples for genetic and proteomic analysis were collected upon ICU admission (henceforth referred to as first post-admission) and at 24 and 48 h post-admission among pTBI participants, while control samples were collected at a single time point (upon admission to the healthcare center). The total blood sampling volume was 2 cc/kg, up to a maximum of 5 ml for children aged 0–4 years and 10 ml for children aged 5–18 years. Red top SST BD Vacutainer® Plus tubes were used for blood collection. Following IRB-approved protocols, we transferred de-identified samples from the University of Florida to the University of Miami for analysis. Serum was stored at –80 °C post-centrifugation in 0.5 μL aliquots. We performed the quantification of inflammasome proteins at Dr. Keane and Dr. de Rivero Vaccari’s lab at the Miami Project to Cure Paralysis, using the Simple Plex Assay on the Ella System.^22^ The assay was analyzed using the Simple Plex Explorer software. The results shown correspond to the mean of each sample run in triplicate. Specifications about the assay properties used in this analysis were the same as described by the authors in a previous publication.^23^ While participants in the TBI cohort underwent three blood draws as previously described, control participants underwent one blood draw to minimize burden and risk in healthy participants.

### Primary outcome

We assessed the association of serum inflammasome protein levels at first post-admission, 24, and 48 h post-admission among pTBI participants, combining all GCS severities into one category, and compared these levels to those in control samples. Our primary outcome was to determine if serum ASC and caspase-1 levels significantly differed between pTBI participants and controls. Additionally, we classified the pTBI cohort based on their GCS scores at admission into mild (GCS 13–15), moderate (GCS 9–12), and severe (GCS 3–8), and compared the serum inflammasome levels of these subgroups to those of control samples.

### Statistical analysis

We considered a sample size of 20 per group based on an alpha (α) of 0.05 (two-tailed) and power (1–*β*) of 0.80. We reported frequencies and percentages for categorical variables. We assessed associations between variables using *χ*^2^ test for categorical variables or Kruskal-Wallis Test for continuous. We presented data as medians with interquartile ranges (IQRs) due to nonparametric distributions of our dataset. We excluded outliers using the Robust Regression and Outlier Removal (ROUT) method. We generated descriptive statistics and tested normality through the Shapiro-Wilk Test. We compared serum inflammasome levels in pTBI participants versus controls using Kruskal-Wallis Test, followed by Dunn’s multiple comparisons test. We set the significance of *p*-values at two-tailed *p* < 0.05 for all statistical tests. To address the variability in non-parametric distributions, we utilized bootstrapping to estimate 95% confidence intervals for the differences in mean ranks between groups. We determined diagnostic accuracy by calculating the Area Under the Receiver Operating Characteristic Curve (AUROC) for sensitivity (SN), specificity (SP), and likelihood ratios (LR), categorizing AUROC values as poor discrimination (<0.7), fair discrimination (0.7–0.8), good discrimination (>0.8–0.9), or excellent discrimination (>0.9). For each first-admission biomarker, we fitted a multivariate linear regression model that included seizure activity, fever at admission, age, sex, Injury Severity Score (ISS), and Pediatric Risk of Mortality (PRISM) score as predictors. For the matched-pair analysis, we used the Wilcoxon signed‑rank test, due to the non-parametric distribution of our sample, to evaluate within‑subject changes in biomarker levels over time. All statistical analyses were conducted in GraphPad Prism version 10 and JMP 18.

## Results

### Cohort characteristics

**Fig. 1:**
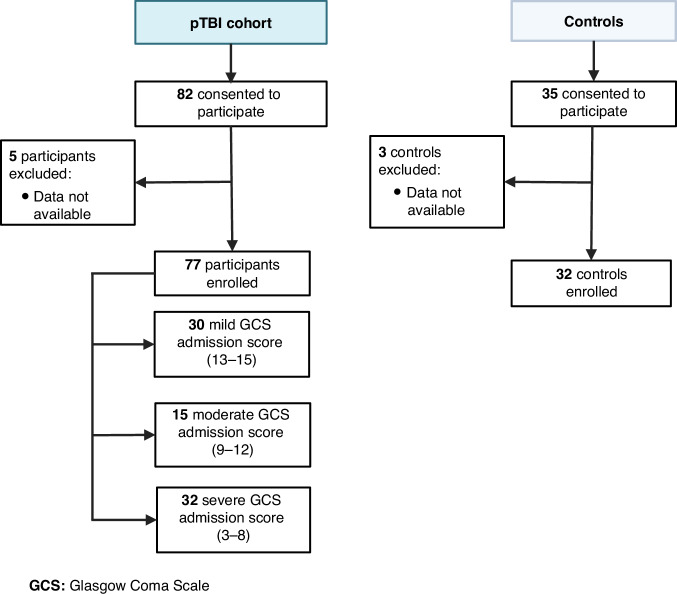
Study workflow. Diagram depicting participant flow from initial recruitment through to final data analysis stages.

We enrolled and consented a total of 82 participants with a clinical diagnosis of pTBI and 35 controls. Due to insufficient data for statistical analysis, we excluded five pTBI participants and three controls. The final analysis included 77 pTBI participants and 31 controls, all with recorded GCS scores upon admission. Additionally, all participants included in the final analysis had follow-up serum data available in ≥1 of our selected timepoints (Fig. 1).

We detailed baseline characteristics of pTBI and control participants in Table 1. Among the pTBI cohort, 30 (39%) presented with mild TBI, 15 (19%) with moderate TBI, and 32 (42%) with severe TBI. No significant differences were observed between the control and pTBI cohorts in terms of age, sex, weight, height, body mass index (BMI), ethnicity, and race. Additional information regarding the initial diagnostic information for the control cohort is provided in Supplementary Table 1. Within the pTBI cohort, PRISM and ISS scores at admission were found to be statistically significant across varying GCS severity levels. Conversely, mechanisms of TBI injury, presence of a comorbidity, or ICU medication usage did not exhibit statistically significant variation across different GCS categories. Although CT-positive findings were more frequent in severe GCS participants, the difference was not statistically significant from the mild and moderate categories.

**Table 1:** Demographic and injury characteristics of pediatric traumatic brain injury (pTBI) subjects categorized by Glasgow Coma Scale (GCS) severity, and controls.

Characteristics	Controls	pTBI Cohort			*p*-value^a^
		(*n* = 77)			
		Mild	Moderate	Severe	
		(GCS 13–15)	(GCS 9–12)	(GCS 3–8)	
	(*n* = 31)	(*n* = 30)	(*n* = 15)	(*n* = 32)	
**Age, median (IQR), y**	6 (9)	8 (11.5)	9 (10)	7 (12.25)	0.86
**0–1**	0	0.9 (0.17)	0.5 (0.5)	0.75 (0.67)	
**2–5**	3.5 (1)	3.3 (3.4)	3 (3)	4 (2)	
**6–11**	8 (3)	8 (3.89)	9 (3)	9 (3.2)	
**12–18**	15 (4)	14 (3)	14.5 (2)	15 (1)	
**Sex, No. (%)**					
**Female**	18 (58.06)	9 (30)	4 (26.67)	11 (34.38)	0.07
**Male**	13 (41.94)	21 (70)	11 (73.33)	21 (65.63)	
**Weight, median (IQR), kg**	26 (28.45)	30 (47.65)	36 (38.5)	33.5 (63.8)	0.98
**Height, median (IQR), cm**	122 (52.5)	121.9 (106.2)	137.2 (58.4)	144.8 (78.1)	0.97
**BMI, median (IQR)**	16 (4.99)	19.27 (5.97)	18.3 (6)	21.35 (8.5)	0.86
**Ethnicity, no. (%)**					
**Hispanic or Latino**	4 (12.9)	5 (16.67)	4 (26.67)	3 (9.38)	0.7
**Not Hispanic or Latino**	27 (87.1)	19 (63.3)	9 (60)	6 (18.8)	
**Choose not to disclose**	0 (0)	6 (20)	2 (13.33)	23 (71.88)	
**Race, No. (%)**					
**White**	21 (67.74)	21 (70)	7 (46.67)	22 (68.75)	0.59
**African American**	10 (35.2526)	6 (20)	5 (33.33)	6 (18.75)	
**Asian**	0 (0)	1 (3.33)	0 (0)	1 (3.13)	
**Unknown**	0 (0)	0 (0)	2 (13.33)	2 (6.25)	
**Unreported**	0 (0)	2 (6.67)	1 (6.67)	1 (3.13)	
**Presence of comorbidities, No. (%)**^**b**^	NA	6 (20)	2 (13.3)	12 (37.5)	0.13
**ICU medication usage, No. (%)**^**c**^					
**Anti-inflammatory**	NA	6 (20)	4 (26.7)	10 (31.3)	0.16
**Anti-seizure**		0	2 (13.3)	8 (25)	
**Sedatives**		10 (33.3)	10 (66.7)	30 (93.8)	
**Mechanism of TBI, No. (%)**					
**Acceleration/Deceleration**	NA	8 (26.66)	6 (40)	15 (46.87)	0.63
**Direct impact**		12 (40)	6 (40)	12 (37.5)	
**Ground-level fall**		1 (3.33)	1 (6.67)	0 (0)	
**Fall from height** > **1 meter (3 ft)**		4 (13.33)	1 (6.67)	1 (3.13)	
**Blow to head**		4 (13.33)	1 (6.67)	3 (9.38)	
**Penetrating head trauma**		1 (3.33)	0 (0)	1 (3.13)	
**ISS, No. (%)**					
**Minor to serious (1–24)**	NA	13 (43.33)	4 (26.67)	2 (6.25)	0.05
**Severe to maximum (25–75)**		8 (26.67)	9 (60)	26 (81.25)	
**Unreported**		9 (30)	2 (13.33)	4 (12.25)	
**PRISM score, no. (%)**					
**0–4**	NA	0 (0)	0 (0)	0 (0)	0.04
**5–9**		10 (33.3)	2 (13.33)	0 (0)	
**10–14**		6 (20)	4 (26.67)	3 (9.37)	
**15–19**		1 (3.33)	0 (0)	5 (15.62)	
**20–24**		0 (0)	0 (0)	3 (9.37)	
**25–29**		0 (0)	0 (0)	2 (6.25)	
**30–34**		0 (0)	0 (0)	0 (0)	
**≥35**		0 (0)	0 (0)	1 (3.125)	
**Unreported**		13 (43.3%)	9 (60)	18 (56.25)	
**Abnormal findings at baseline CT, no. (%)**					
**CT Positive**^**b**^	NA	10 (33.33)	7 (46.66)	20 (62.5)	0.39
**Extracranial Injury**		16 (53.3)	5 (33.3)	13 (40.6)	
**Intracranial Injury**		14 (46.7)	10 (66.7)	19 (59.4)	
**CT Negative**		20 (66.67)	8 (53.33)	12 (37.5)	

*BMI* Body Mass Index, *cm* centimeters, *CT* computed tomography, *GCS* Glasgow Coma Scale, *ISS* Injury Severity Score, *kg* kilograms, *PRISM* Pediatric Risk of Mortality Score, *pTBI* pediatric traumatic brain injury, *SD* standard deviation, *TBI* traumatic brain injury, *y* years.

^a^*p*-value obtained from χ^2^ test or Kruskal-Wallis Test.

^b^Comorbidities extracted for this analysis include diagnoses of cardiac arrest, hypothermia, and/or seizures.

^c^Data reported and analysis performed only to the participants who received the corresponding medication. Because the counts are limited to these subgroups, the summed *n* values do not equal the total cohort size.

### Serum inflammasome profiles at first, 24, and 48 h post-admission in pTBI participants versus controls

**Fig. 2:**
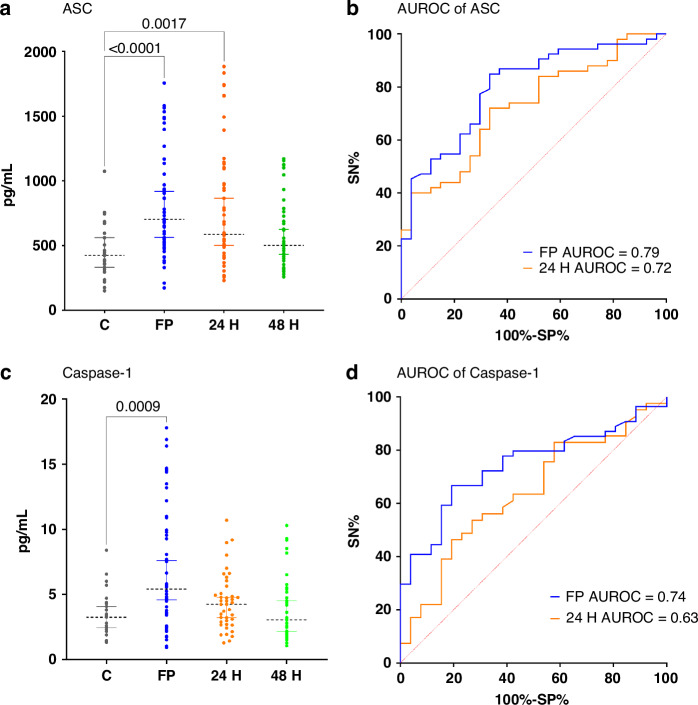
Serum concentrations of inflammasome proteins in pTBI participants at first post-admission, 24, and 48 h post-injury versus controls. Dot plots depict statistically significant differences between serum inflammasome protein levels (pg/mL) in pTBI participants collected at first post-admission (FP), 24, and 48 h post-admission versus control samples. Corresponding ROC Curves of statistically significant associations are presented next to each plot. **a** ASC in pTBI samples taken at first post-admission (FP), 24H, and 48H post-admission versus control samples at first post-admission across combined GCS categories. **b** Receiver Operating Characteristics (ROC) of ASC at FP and 24H post injury across injury severity compared to controls. **c** Caspase-1 in pTBI samples taken at first post-admission (FP), 24H, and 48H post-admission versus control samples at first post-admission in combined GCS categories. **d** ROC of caspase-1 at FP and 24H post injury severity compared to controls. Dots represent individual values of serum samples (pg/mL); error bars depict the 5th and 95th percentiles. The dotted line represents the median from the dataset. Numbers above connecting lines represent statistically significant *p* values obtained by Dunn’s multiple comparisons test. ASC indicates Apoptosis-associated speck-like protein containing a caspase recruitment domain; AUROC, Area Under the Receiver Operator Characteristics curve; *C* controls, *FP* first post-admission, *H* hours, *SN* sensitivity, *SP* specificity.

We observed higher levels of ASC at first post-admission in the pTBI group compared to the control group (*p* < 0.0001, mean rank difference (MD) of 51.36 pg/mL, 95% CI: 50.50, 54.76 pg/mL, and an AUROC of 0.79. At 24 h, ASC levels remained significantly higher compared to controls (*p* = 0.0017, MD 40.75 pg/mL, 95% CI: 38.38, 42.86 pg/mL), with an AUROC of 0.72. Similarly, Caspase-1 levels were higher at first post-admission in the pTBI group compared to controls (*p* = 0.0010, MD 60.52 pg/mL, 95% CI: 36.48, 41.72 pg/mL), with an AUROC of 0.74. AUROC values for all comparisons performed in this analysis demonstrate fair discrimination between pTBI and control participants (Fig. 2). Additional details of the individual analysis of each of the serum proteins are presented in Table 2.

**Table 2:** Characteristics of serum inflammasome protein samples at first 24, and 48 hours (H) post-admission in pTBI participants across injury severity categories by GCS vs. control samples^a^.

Inflammasome proteins	Timepoints	*p*-value^b^	Mean rank difference^b,c^	95% CI^c^	AUROC	SE	Additional values	
**ASC**	First post-admission	<0.0001	51.36	50.50, 54.76	0.79	0.053	Cutoff^c^	>758.0
							SN	45.28%
							SP	96.30%
							LR	12.23
	24H post-admission	0.0017	40.75	38.38, 42.86	0.72	0.058	Cutoff^c^	>766
							SN	40%
							SP	96.30%
							LR	10.80
**Caspase-1**	First post-admission	0.0010	60.52	36.48, 41.72	0.74	0.051	Cutoff^c^	>6.61
							SN	40.74%
							SP	96.15%
							LR	10.59

*AUROC* area under the receiver operator characteristics curve, *CI* confidence interval, *GCS* Glasgow Coma Scale, *IQR* interquartile range, *LR* likelihood ratio, *PTBI* pediatric traumatic brain injury, *SN* sensitivity, *SP* specificity, *SE* standard error.

^a^Only statistically significant results are presented.

^b^Results from Dunn’s multiple comparison test results.

^c^Values given in pg/mL.

### Serum inflammasome profiles at first post-admission, 24, and 48 h post-admission in pTBI participants categorized by admission GCS severity versus controls

**Fig. 3:**
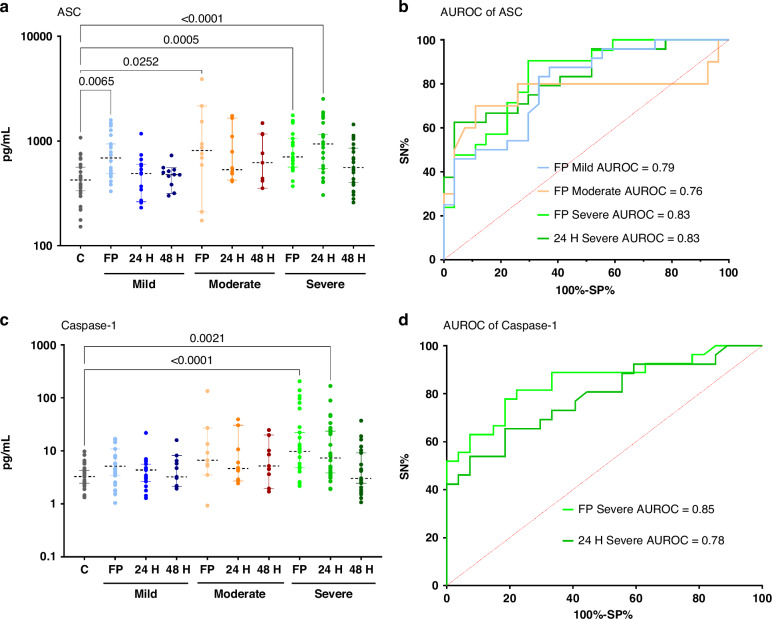
Serum concentration of inflammasome proteins in post-injury pTBI participants versus controls classified by severity levels determined by admission Glasgow Coma Scale scores. Dot plots depict statistically significant differences between serum inflammasome protein levels (pg/mL) in pTBI participants TBI collected at first post-admission (FP), 24, and 48 h post-admission versus control samples, categorized by GCS severity: Mild, Moderate, and Severe. Corresponding ROC Curves of statistically significant associations are depicted next to each box plot. **a** ASC in pTBI samples taken at first post-admission (FP), 24H, and 48H post-admission versus control samples at first post-admission classified by GCS severity categories. **b** Receiver Operating Characteristics (ROC) of ASC at FP across injury severity and at 24H post injury in severe TBI compared to controls. **c** Caspase-1 in pTBI samples taken at first post-admission (FP), 24H, and 48H post-admission versus control samples at first post-admission classified by GCS severity categories. **d**ROC of caspase-1 at FP and 24H post injury severity compared to controls. Dots represent individual values of serum samples (pg/mL); error bars depict the 5th and 95th percentiles. The dotted line represents the median from the dataset. Numbers above connecting lines represent statistically significant *p* values obtained by Dunn’s multiple comparisons test. ASC apoptosis-associated speck-like protein containing a caspase recruitment domain; *AUROC* area under the receiver operator characteristics curve, *C* controls, *FP* first post-admission, *H* hours, *SN* sensitivity, *SP* specificity.

A summary of the findings from the analysis of pTBI samples categorized by GCS severity versus control samples is presented in Table 3. At first post-admission, ASC levels were significantly higher across all three GCS categories (mild, moderate, and severe), with the most pronounced differences observed in the severe GCS category (*p* = 0.0005, MD 59.78 pg/mL, 95% CI 56.14, 60.40 pg/mL), with an AUROC of 0.83. ASC levels were also elevated in samples taken 24 h post-admission in the severe category (*p* < 0.0001, MD 62.53 pg/mL, 95% CI 60.61, 65.49 pg/mL), with an AUROC of 0.83. Caspase-1 levels at first post-admission and 24 h post-admission were significantly increased in the severe category in pTBI participants compared to controls. The most significant association was observed in samples taken at first post-admission (*p* < 0.0001, MD 64.5 pg/mL, 95% CI 60.87, 66.17 pg/mL), with an AUROC of 0.85, indicating significant differences and discrimination ability between pTBI participants and controls (Fig. 3).

**Table 3:** Characteristics of serum inflammasome protein samples at first 24, and 48 hours (H) post-admission in pTBI participants classified by severity categories based on GCS, and control samples^a^.

Inflammasome proteins	Timepoints	GCS severity	*p*-value^b^	Mean Rank difference^b,c^	95% CI^c^	AUROC	SE	Additional values	
**ASC**	First post-admission	Mild	0.0065	48.07	45.19, 50.71	0.79	0.061	Cutoff^c^	>769.5
								SN	45.83%
								SP	96.30%
								LR	12.38
		Moderate	0.0252	56.06	53.78, 60.52	0.76	0.117	Cutoff^c^	>811.0
								SN	50%
								SP	96.30%
								LR	13.5
		Severe	0.0005	59.78	56.14, 60.40	0.83	0.056	Cutoff^c^	>758.0
								SN	47.62%
								SP	96.30%
								LR	12.86
	24H post-admission	Severe	<0.0001	62.53	60.61, 65.49	0.83	0.05285	Cutoff^c^	>766.0
								SN	62.5%
								SP	96.30%
								LR	16.88
**Caspase-1**	First post-admission	Severe	<0.0001	64.5	60.87, 66.17	0.85	0.053	Cutoff^c^	>8.975
								SN	55.56%
								SP	96.30%
								LR	15
	24H post-admission	Severe	0.0021	53.65	50.18, 55.24	0.78	0.06327	Cutoff^c^	>8.690
								SN	46.15%
								SP	96.30%
								LR	12.46

*AUROC* area under the receiver operator characteristics curve, *CI* confidence interval, *GCS* Glasgow Coma Scale, *IQR* interquartile range, *LR* likelihood ratio, *PTBI* pediatric traumatic brain injury, *SN* sensitivity, *SP* specificity, *SE* standard error.

^a^Only statistically significant results are presented.

^b^Results from Dunn’s multiple comparison test results.

^c^Values given in pg/mL.

### Multivariate analyses

We applied a multivariate linear regression to the first post-admission biomarker concentrations, using seizure activity, peripheral trauma, fever during admission, age, sex, ISS, and PRISM score as predictors. None of these variables was independently associated with biomarker levels (all *p* > 0.10), indicating that comorbid factors did not meaningfully alter marker concentrations in our cohort. Within-subject comparisons showed a statistically significant decline in ASC serum levels over time, with higher concentrations observed at the first post-admission and 24 h timepoints compared to 48 h. Specifically, ASC levels declined from first to 48 h (*p* = 0.001, MD−812 pg/mL, 95% CI: −1366.2, −258.15 pg/mL) and from 24 to 48  (*p* = 0.0002, MD−507 pg/mL, 95% CI: −912.96, −101.04 pg/mL). Caspase-1 levels similarly declined from first to 48 h (*p* = 0.0008, MD−24.69 pg/mL, 95% CI: −48.91, −0.47 pg/mL) and from 24 to 48 h (*p* = 0.0004, MD−13.31 pg/mL, 95% CI: −28.50, 1.8 pg/mL). Detailed values and matched-pair plots are presented in Supplementary Fig. 1.

## Discussion

In this prospective observational cohort study, we found that ASC serum levels were elevated in pTBI patients at both first post-admission and 24 h post-admission across all GCS categories compared to controls. Similarly, caspase-1 levels were also increased at first post-admission for all GCS categories, demonstrating a consistent inflammatory response in the serum. Further analysis by GCS severity showed that ASC levels remained persistently elevated across mild, moderate, and severe categories at both first post-admission and 24 h. Our findings suggest that ASC and caspase-1 levels may correspond with the degree of neurologic injury, supporting their potential role as markers of clinical severity. While this study was not designed to assess long-term outcomes, these biomarkers may also hold prognostic value and highlight the contribution of NLRP3-mediated inflammation in the pathophysiology of pediatric TBI. Further longitudinal and mechanistic studies are warranted to clarify their utility.

Our results reflect similar findings previously published in animal models and adult TBI research. A study performed in adult patients found that ASC levels rise promptly following TBI, both in serum and CSF samples, compared to controls.^24^ Additionally, our findings showing elevated caspase-1 levels at first post-admission in combined GCS severities, as well as in the standalone severe GCS category, are consistent with adult TBI research that associates higher levels of caspase-1 in CSF samples with clinical indicators of brain injury severity in the ICU, and associated poor neurologic outcomes.^12,25–28^

Despite the complexity and variability of TBI, current methods for defining and assessing its severity in both adult and pediatric populations remain inadequate. Historically, early assessment of TBI has been based primarily on clinical features and neuroimaging. Although imaging studies such as CT have improved our understanding and management of TBI, they are most beneficial for the small subset of patients requiring neurosurgical interventions, as routine imaging is not recommended for mild TBI cases.^29,30^ Prediction models that combine variables assessed during the acute phase have only achieved modest accuracy in predicting long-term outcomes.^31^

This issue is particularly significant given that over 90% of TBI cases are classified as mild, including concussions.^29^ While most patients recover, some experience prolonged symptoms and increased post-traumatic risks, leading to a cumulative social burden and treatment costs comparable to moderate and severe TBIs.^32^ An interesting finding in our results is that in mild pTBI cases from our cohort, ASC levels increased in early first post-admission samples in the mild GCS category, albeit with moderate sensitivity. This is especially important as the diagnosis and assessment of mild pTBI is usually a challenging task. Existing guidelines suggest that a diagnostic approach should consist of a combination of clinical evaluation, symptom assessment, and selective use of imaging and cognitive testing tools.^33^

Current knowledge of the role of neuroinflammatory responses indicates they play a crucial role in the acute and subacute stages of TBI. Initially, the inflammatory response is thought to be a protective mechanism aimed at limiting tissue damage, clearing debris, and promoting cell repair. However, excessive or prolonged inflammation can lead to secondary injury, exacerbating neuronal damage and contributing to long-term neurological deficits.^6,34^

In pediatric TBI, the developing brain may be particularly vulnerable to the effects of inflammation. Compared to adults, pediatric neurological injury leads to an increased propensity for neuronal apoptosis, age-dependent parameters for cerebral blood flow and metabolism, an increased likelihood of early post-traumatic seizures, and altered neuroplasticity during recovery from injury.^35^ Consequently, ongoing developmental processes such as synaptogenesis and myelination can be disrupted, leading to varying levels of neurologic outcomes.^36^ As such, a controlled neuroinflammatory process seems to be the goal to achieve in future research models to mitigate the deleterious effects of excessive neuroinflammatory processes post-pTBI.

The results of our study open the possibility of targeting the NLRP3 inflammasome and proinflammatory cytokines in early TBI management.^37^ Generalized anti-inflammatory therapeutic approaches for the management of TBI, such as systemic corticosteroids and hypothermia, have failed to demonstrate benefits—and were even deleterious—in multicenter clinical trials.^38,39^ As a result, the development of inflammasome-specific inhibitors is an emerging area of research that shows promise. Inhibitors like MCC950 and other agents such as OLT1177 and Oridonin, as well as targeted therapies like IC 100, show potential in improving neurological outcomes in animal models.^40–44^ The clinical implications of these studies, including the viability of these treatments in pTBI, are areas for future research efforts.

This study enhances our understanding of the role of NLRP3 inflammasome activation in pediatric TBI by profiling serum levels of these proteins in the acute phase of TBI and examining their clinical correlation with injury severity. Future research should further explore the relationship between serum inflammasome protein levels and specific neuronal injury biomarkers. Current studies in pTBI have identified that biomarkers such as glial fibrillary acidic protein (GFAP), neuron-specific enolase (NSE), ubiquitin C-terminal hydrolase-L1 (UCH-L1), S100 calcium-binding protein B (S100B), osteopontin, and tau protein are significantly elevated in children with severe TBI, highlighting their potential as indicators of injury severity and tools for disease stratification.^45,46^ Integrating these biomarkers with established brain injury severity prediction tools could aid in developing accurate, non-invasive models for predicting neurological outcomes post-TBI, especially in mild cases where severity assessment is challenging. Such models could provide valuable insights for clinicians and caregivers regarding post-TBI prognosis. Additionally, this study lays the groundwork for future investigations into the role of these proteins in therapeutic strategies for pTBI, specifically targeting inflammasomes and related proinflammatory cytokines.

Some limitations to consider in this study include a relatively small sample size, non-randomized methods, and reliance on a single tool for severity assessment for pragmatic purposes. Additionally, the study’s 48 h temporal profile restricts insights into inflammasome protein trends over time, highlighting the need for future research to explore longer timeframes for a comprehensive understanding of inflammatory responses. Given the ubiquitous expression of NLRP3 proteins in different tissues, polytrauma and secondary insults, particularly after severe TBI, may confound increased central nervous system and systemic inflammasome protein expression. However, it is important to note that both preclinical and clinical studies indicate that secondary insults shift the immune response towards anti-inflammatory cytokines like IL-10, suggesting that a systemic pro-inflammatory state may not significantly alter local neuroinflammatory responses.^47–49^ Nonetheless, this consideration should be addressed in further studies to strengthen the validity of the results.

Another limitation concerns our nomenclature for timing relative to injury. Because the exact injury time was inconsistently documented, given the nature of the disease presentation, we anchored all biomarker sampling to the moment of hospital admission; unmeasured variation in the interval between injury and blood draw may therefore introduce residual temporal confounding that we could not fully address in our analyses. Additionally, although the study was not powered to adjust for the individual effects of each medication class, we acknowledge their potential confounding influence on biomarker levels and plan to examine these effects in future analyses or sensitivity models when feasible.

Finally, the relatively healthy control group, lacking ICU admission or documented PRISM scores, constituted a limitation because we could not fully evaluate how illness severity might have influenced NLRP3 levels. Future research involving a more critically ill control cohort, with documented severity scores, would enable more robust comparisons of NLRP3 expressions across different clinical contexts.

## Supplementary information

Revised-Supplemental_Materials

## Data Availability

The datasets generated during and/or analyzed during the current study are available from the corresponding author on reasonable request, after appropriate documentation is completed by the relevant entities.
