# Supplementary material for: Association of serum inflammasome proteins and pediatric traumatic brain injury severity
Source: Pediatr Res. 2025 Sep 26;99(4):1494–503. doi: 10.1038/s41390-025-04410-5 (PMC13102685; doi:10.1038/s41390-025-04410-5)

Supplemental Materials

Supplemental Table S1. Diagnosis Descriptions of the Control Cohort.			
**Categories**	**ICD-10 Code**	**Admission Diagnoses**	**N=31**
Infectious	H66.90	Acute otitis media, unspecified, unspecified ear	1
	L03.031	Cellulitis of toe	1
	L03.90	Cellulitis, unspecified	1
	B34.9	Viral infection, unspecified	2
	K35.80	Unspecified acute appendicitis	2
Non-Infectious	N28.1	Cyst of kidney, acquired	1
	N13.1	Hydronephrosis with ureteropelvic junction obstruction	1
	N20.0	Kidney stone	1
	S72.009A	Unspecified fracture of unspecified femur, initial encounter for closed fracture	1
	Z48.02	Encounter for removal of sutures following surgery	1
	I10	Essential (primary) hypertension	1
	I27.20	Pulmonary hypertension, unspecified	1
	S61.219A	Laceration without foreign body of unspecified finger without damage to nail, initial encounter	1
	S99.819A	Other specified injuries of unspecified ankle and foot, initial encounter	1
	M65.9	Tenosynovitis, unspecified	1
	K50.919	Crohn's disease, unspecified, with exacerbation	1
	R11.2	Nausea with vomiting, unspecified	1
	M70.71	Other bursitis of hip	1
	K02.9	Dental caries, unspecified	9
	Q74.9	Congenital malformations of limb(s), unspecified	1
	K22.2	Esophageal obstruction	1
International Classification of Diseases (ICD) coder			

Supplemental Table S2. STROBE Statement—checklist of items that should be included in reports of observational studies

	Item No.	Recommendation	Page No.	Relevant text from manuscript
**Title and abstract**	1	(*a*) Indicate the study's design with a commonly used term in the title or the abstract	1	Association of Serum Inflammasome Proteins and Pediatric Traumatic Brain Injury Severity
		(*b*) Provide in the abstract an informative and balanced summary of what was done and what was found	3	Background: Pediatric traumatic brain injury (pTBI) often leads to cognitive, behavioral, and motor impairments. NLRP3 inflammasome proteins, such as ASC and caspase-1, may serve as biomarkers for TBI severity due to their role in neuroinflammation. This study aimed to assess the association between serum ASC and caspase-1 levels and TBI severity in pediatric patients.<br><br>Methods: Serum samples were collected at pediatric intensive care unit (ICU) admission (first post-admission), and at 24 and 48 hours post-admission, from TBI participants aged 28 days to 18 years and from demographically matched controls. TBI severity was assessed using the Glasgow Coma Scale (GCS).<br><br>Results: We analyzed samples from 77 pTBI patients and 31 controls. ASC levels were significantly higher across all GCS categories, with the most pronounced differences in the severe category at first post-admission (p= 0.0005, AUROC 0.83) and 24 hours post-admission (p< 0.0001, AUROC 0.83). Caspase-1 levels were significantly elevated in the severe category, particularly at first post-admission (p< 0.0001, AUROC 0.85).<br><br>Discussion: Elevated ASC and caspase-1 levels, especially in severe pTBI cases, suggest their potential as biomarkers for TBI severity. These findings emphasize the role of inflammasome proteins in post-TBI neuroinflammation and support further research into targeted therapies for pediatric TBI.
**Introduction**				

Background/rationale	2	Explain the scientific background and rationale for the investigation being reported	4	In clinical traumatic brain injury (TBI) research, NLRP3 inflammasome-associated proteins have been evaluated as potential injury biomarkers in cerebrospinal fluid (CSF) and serum samples of adult patients<br><br>In pediatric patients, however, these proteins have only been studied in CSF by a single-centered study, where higher levels of NLRP3 proteins were found in pTBI patients' samples compared to controls across all time points.<br><br>Nevertheless, a gap in current clinical knowledge exists regarding post-TBI inflammasome serum profiles and their association with pTBI severity.
Objectives	3	State specific objectives, including any prespecified hypotheses	5	This study aimed to evaluate the association of serum levels of NLRP3 inflammasome, specifically apoptosis-associated speck-like protein containing a caspase recruitment domain (ASC) and caspase-1, as candidate biomarkers of neuroinflammatory activation following pediatric TBI. These inflammasome-related proteins may reflect early innate immune activation and serve both as potential indicators of injury severity and as exploratory mechanistic markers of NLRP3 pathway involvement. We hypothesized that serum levels of these proteins will be elevated correspondingly to pTBI severity in participants versus control subjects.
**Methods**				
Study design	4	Present key elements of study design early in the paper	5	Prospective observational cohort study
Setting	5	Describe the setting, locations, and relevant dates, including periods of recruitment, exposure, follow-up, and data collection	5	Pediatric intensive care units (ICUs) of two academic medical centers in Florida, USA, from February 2017 to June 2023.
Participants	6	(*a*) *Cohort study*—Give the eligibility criteria, and the sources and methods of selection of participants. Describe methods of follow-up<br>*Case-control study*—Give the eligibility criteria, and the sources and methods of	5,6	We recruited eligible participants aged 28 days to 18 years who were admitted or transferred to the pediatric intensive care units (ICUs) of the medical centers. In accordance with our Institutional Review Board (IRB) approved protocol for University of Florida (IRB201600237) and the University of Miami (IRB 20210352), we obtained written informed consent from a parent or guardian for participants under 18 years, while participants who were 18 years old provided consent on their own behalf.

		case ascertainment and control selection. Give the rationale for the choice of cases and controls<br>*Cross-sectional study*—Give the eligibility criteria, and the sources and methods of selection of participants		We approached eligible participants or their legal representatives immediately upon admission at the University of Miami, and within 72 hours after admission at the University of Florida, by trained research staff. Recruitment was conducted in a manner to minimize selection bias, ensuring that all eligible patients during the study period were screened and approached systematically based on their admission time and clinical status.<br>Inclusion criteria was a clinical diagnosis of TBI upon arrival. We defined TBI as a brain injury caused by an outside force.15 We excluded participants with psychiatric disorders exhibiting severe features as per the Diagnostic and Statistical Manual of Mental Disorders (Fifth Edition), pregnancy, or insufficient data for statistical analysis.16 Patients with known systemic infections or diagnosed autoimmune conditions were also excluded.<br><br>Controls were selected if they fulfilled similar demographic characteristics as the selected participants, and were recruited from the pediatric emergency department, pediatric in-patient floor, and outpatient sedation services from both medical centers. Exclusion criteria for controls included a history of pTBI, pregnancy, acute neurologic dysfunction or psychiatric disorders exhibiting severe features. 17 We assessed TBI severity using a modified pediatric version of the Glasgow Coma Scale (GCS) for participants aged 2 years or younger or who were pre-verbal, and we used the standard GCS upon admission for both participants and controls. We used the lowest GCS score recorded for each participant for the final analysis.
		(*b*) *Cohort study*—For matched studies, give matching criteria and number of exposed and unexposed<br>*Case-control study*—For matched studies, give matching criteria and the number of controls per case	N/A	*Not a matched cohort study, but controls with similar demographic characteristics were selected.
Variables	7	Clearly define all outcomes, exposures, predictors, potential confounders, and effect modifiers. Give diagnostic criteria, if applicable	6,7	Our primary outcome was to determine if serum ASC and caspase-1 levels significantly differed between pTBI participants and controls. Additionally, we classified the pTBI cohort based on their GCS scores at baseline into mild (GCS 13-15), moderate (GCS 9-12), and severe (GCS 3-8), and compared the serum inflammasome levels of these subgroups to those of control samples.

Data sources/ measurement	8*	For each variable of interest, give sources of data and details of methods of assessment (measurement). Describe comparability of assessment methods if there is more than one group	5,6	We obtained written consent from participants, controls, or their legal representatives to collect blood samples and data from electronic health records (EHR).<br><br>We collected TBI-Common Data Elements (TBI-CDE), caregiver-reported demographic information, and detailed clinical assessments, as well as a seven-day compilation of relevant critical care data points extracted from EHRs.18 All drugs administered from emergency-department arrival through the final blood draw were abstracted from EHR. Medication classes specifically tracked included antiseizure, anti-inflammatory (NSAID or corticosteroid), sedative-anxiolytic, and immunomodulatory agents.<br><br>We followed the TBI-CDE Biospecimens and Biomarkers Working Group Consensus guidelines for serum preparation.<br><br>Blood samples for genetic and proteomic analysis were collected upon ICU admission (henceforth referred to as baseline) and at 24 and 48 hours post-admission among pTBI participants, while control samples were collected at a single time point (upon admission to healthcare center).
Bias	9	Describe any efforts to address potential sources of bias		Recruitment was conducted in a manner to minimize selection bias, ensuring that all eligible patients during the study period were screened and approached systematically based on their admission time and clinical status.
Study size	10	Explain how the study size was arrived at	7	We considered a sample size of 20 per group based on an alpha (α) of 0.05 (two-tailed) and power ($1 - \beta$) of 0.80.

Continued on next page

Quantitative variables	11	Explain how quantitative variables were handled in the analyses. If applicable, describe which groupings were chosen and why	6,7	We assessed TBI severity using the Glasgow Coma Scale (GCS) upon admission for both participants and controls. We used the lowest GCS score recorded for each participant for the final analysis.<br><br>We assessed the association of serum inflammasome protein levels at baseline, 24, and 48 hours post-consent among pTBI patients, combining all GCS severities into one category, and compared these levels to those in control samples<br><br>We classified the pTBI cohort based on their GCS scores at baseline into mild (GCS 13-15), moderate (GCS 9-12), and severe (GCS 3-8), and compared the serum inflammasome levels of these subgroups to those of control samples.
Statistical methods	12	(*a*) Describe all statistical methods, including those used to control for confounding	7	We considered a sample size of 20 per group based on an alpha (α) of 0.05 (two-tailed) and power (1 – β) of 0.80. We reported frequencies and percentages for categorical variables. We assessed associations between variables using χ2 test for categorical variables or Kruskal-Wallis Test for continuous. We presented data as medians with interquartile ranges (IQRs) due to nonparametric distributions of our dataset. We excluded outliers using the Robust Regression and Outlier Removal (ROUT) method. We generated descriptive statistics and tested normality through the Shapiro-Wilk Test. We compared serum inflammasome levels in pTBI participants versus controls using Kruskal-Wallis Test, followed by Dunn's multiple comparisons test. We set the significance of p-values at two-tailed p< 0.05 for all statistical tests. To address the variability in non-parametric distributions, we utilized bootstrapping to estimate 95% confidence intervals (CIs) for the differences in mean ranks between groups. We determined diagnostic accuracy by calculating the Area Under the Receiver Operating Characteristic Curve (AUROC) for sensitivity (SN), specificity (SP), and likelihood ratios (LR), categorizing AUROC values as poor discrimination (<0.7), fair discrimination (0.7–0.8), good discrimination (>0.8–0.9), or excellent discrimination (>0.9). We also calculated predictive values and assay accuracy. For each baseline biomarker we fitted a multivariate linear regression model that included seizure activity, fever at admission, age, sex, Injury Severity Score (ISS), and PRISM score as predictors. We used the Wilcoxon signed-rank test, due to the non-parametric distribution of our sample, to evaluate within-subject changes in biomarker levels over time. We conducted all statistical analyses using GraphPad Prism version 10 and JMP 18.
		(*b*) Describe any methods used to examine	7	We compared serum inflammasome levels in pTBI participants versus controls using Kruskal-Wallis

		subgroups and interactions		Test, followed by Dunn's multiple comparisons test.
		(*c*) Explain how missing data were addressed	N/A	N/A
		(*d*) *Cohort study*—If applicable, explain how loss to follow-up was addressed<br>*Case-control study*—If applicable, explain how matching of cases and controls was addressed<br>*Cross-sectional study*—If applicable, describe analytical methods taking account of sampling strategy	N/A	N/A
		(*e*) Describe any sensitivity analyses	N/A	N/A
**Results**				
Participants	13*	(a) Report numbers of individuals at each stage of study—eg numbers potentially eligible, examined for eligibility, confirmed eligible, included in the study, completing follow-up, and analysed	7	We enrolled and consented a total of 82 participants with a clinical diagnosis of pTBI and 35 controls.
		(b) Give reasons for non-participation at each stage	7	Due to insufficient data for statistical analysis, we excluded 5 patients and 4 controls.
		(c) Consider use of a flow diagram	7	**Figure 1**
Descriptive data	14*	(a) Give characteristics of study participants (eg demographic, clinical, social) and information on exposures and potential confounders	7	No significant differences were observed between the control and pediatric traumatic brain injury (pTBI) cohorts in terms of age, sex, weight, height, body mass index (BMI), ethnicity, and race.
		(b) Indicate number of participants with missing data for each variable of interest	7	The final analysis included 77 patients and 31 controls, all with recorded GCS score upon admission.
		(c) *Cohort study*—Summarise follow-up time (eg, average and total amount)	7	Additionally, all patients included in the final analysis had follow-up serum data available in ≥ 1 of our selected timepoints.
Outcome data	15*	*Cohort study*—Report numbers of outcome events or summary measures over time	7	During the 48-hour follow-up period, among the pTBI cohort, 30 (39%) presented with mild TBI, 15 (19%) with moderate TBI, and 32 (42%) with severe TBI.

		Case-control study—Report numbers in each exposure category, or summary measures of exposure	N/A	N/A
		Cross-sectional study—Report numbers of outcome events or summary measures	N/A	N/A
Main results	16	(*a*) Give unadjusted estimates and, if applicable, confounder-adjusted estimates and their precision (eg, 95% confidence interval). Make clear which confounders were adjusted for and why they were included	8	We observed higher levels of ASC at baseline in the pTBI group compared to the control group ($p < 0.0001$, with a mean rank difference (MD) of 51.36 pg/mL, 95% CI: 50.50, 54.76 pg/mL, and an area under the curve (AUROC) of 0.79. At 24 hours, ASC levels remained significantly higher compared to controls ($p = 0.0017$, MD 40.75 pg/mL, 95% CI: 38.38, 42.86 pg/mL), with an AUROC of 0.72. Similarly, Caspase-1 levels were higher at baseline in the pTBI group compared to controls ($p = 0.0010$, MD 60.52 pg/mL, 95% CI: 36.48, 41.72 pg/mL), with an AUROC of 0.74. AUROC values for all comparisons performed in this analysis demonstrates fair discrimination between pTBI and control participants.
		(*b*) Report category boundaries when continuous variables were categorized	8	Additional details of the individual analysis of each of the serum proteins are presented in Table 2.
		(*c*) If relevant, consider translating estimates of relative risk into absolute risk for a meaningful time period	N/A	N/A

Continued on next page

Other analyses	17	Report other analyses done—eg analyses of subgroups and interactions, and sensitivity analyses	8,9,10	A summary of the findings from the analysis of pTBI samples categorized by GCS severity versus control samples is presented in Table 3.<br><br>At baseline, ASC levels were significantly higher across all three GCS categories (mild, moderate, and severe), with the most pronounced differences observed in the severe GCS category (p= 0.0005, MD 59.78 pg/mL, 95% CI 56.14, 60.40pg/mL). The AUROC of 0.83 indicates good discrimination between the levels of this protein in serum samples at baseline of pTBI cohorts admitted with a severe GCS and controls. ASC levels were also elevated in samples taken 24 hours post-injury in the severe category (p< 0.0001, MD 62.53 pg/mL, 95% CI 60.61, 65.49 pg/mL), with an AUROC of 0.83. Caspase-1 levels at baseline and 24 hours post-injury were significantly increased in the severe category in pTBI participants compared to controls. The most significant association was observed in samples taken at baseline (p< 0.0001, MD 64.5 pg/mL, 95% CI 60.87, 66.17 pg/mL), with an AUROC of 0.85, indicating significant differences and discrimination ability between pTBI participants and controls.<br><br>We applied a multivariate linear regression to baseline biomarker concentrations, using seizure activity, fever during admission, age, sex, Injury Severity Score (ISS), and PRISM score as predictors. None of these variables independently associated with biomarker levels (all p > 0.10), indicating that comorbid factors did not meaningfully alter marker concentrations in our cohort. . Within-subject comparisons showed a statistically significant decline in ASC serum levels over time, with higher concentrations observed at the first post-admission and 24-hour timepoints compared to 48 hours. Specifically, ASC levels declined from first to 48 hours (p= 0.001, MD −812 pg/mL, 95% CI: −1366.2, −258.15 pg/mL) and from 24 to 48 hours (p= 0.0002, MD −507 pg/mL, 95% CI: −912.96, −101.04 pg/mL). Caspase-1 levels similarly declined from first to 48 hours (p= 0.0008, MD −24.69 pg/mL, 95% CI: −48.91, −0.47 pg/mL) and from 24 to 48 hours (p= 0.0004, MD −13.31 pg/mL, 95% CI: −28.50, 1.8 pg/mL). Detailed values and matched-pair plots are presented in Supplemental Figure 1.
**Discussion**				
Key results	18	Summarise key results with reference to study objectives	10	In this prospective observational cohort study, we found that ASC serum levels were elevated in pTBI patients at both first post-admission and 24 hours post-admission across all GCS categories compared to controls. Similarly, caspase-1 levels were also increased at first post-admission for all GCS categories, demonstrating a consistent inflammatory response in the serum. Further analysis by GCS severity showed that ASC levels remained persistently elevated across mild, moderate, and severe categories at both first post-admission and 24 hours. Our findings suggest that ASC and Caspase-1 levels may correspond with the degree of neurologic injury, supporting their potential role

				as markers of clinical severity. While this study was not designed to assess long-term outcomes, these biomarkers may also hold prognostic value and highlight the contribution of NLRP3-mediated inflammation in the pathophysiology of pediatric TBI. Further longitudinal and mechanistic studies are warranted to clarify their utility.
Limitations	19	Discuss limitations of the study, taking into account sources of potential bias or imprecision. Discuss both direction and magnitude of any potential bias	12,13	Some limitations to consider in this study include a relatively small sample size, non-randomized methods, and reliance on a single tool for severity assessment for pragmatic purposes. Additionally, the study's 48-hour temporal profile restricts insights into inflammasome protein trends over time, highlighting the need for future research to explore longer timeframes for a comprehensive understanding of inflammatory responses. Given the ubiquitous expression of NLRP3 proteins in different tissues, polytrauma and secondary insults, particularly after severe TBI, may confound increased central nervous system and systemic inflammasome protein expression. In our study, we did not control for secondary insults or polytrauma in our pTBI participants. However, it is important to note that both preclinical and clinical studies indicate that secondary insults shift the immune response towards anti-inflammatory cytokines like IL-10, suggesting that a systemic pro-inflammatory state may not significantly alter local neuroinflammatory responses. 44-46 Nonetheless, this consideration should be addressed in further studies to strengthen the validity of the results.<br><br>Another limitation concerns our nomenclature for timing relative to injury. Because the exact injury time was inconsistently documented, we anchored all biomarker sampling to the moment of hospital admission; unmeasured variation in the interval between injury and blood draw may therefore introduce residual temporal confounding that we could not fully address in our analyses. Additionally, although the study was not powered to adjust for the individual effects of each medication class, we acknowledge their potential confounding influence on biomarker levels and plan to examine these effects in future analyses or sensitivity models when feasible.<br><br>Finally, the relatively healthy control group, lacking ICU admission or documented PRISM scores, constituted a limitation because we could not fully evaluate how illness severity might have influenced NLRP3 levels. Future research involving a more critically ill control cohort, with documented severity scores, would enable more robust comparisons of NLRP3 expressions across different clinical contexts.

Interpretation	20	Give a cautious overall interpretation of results considering objectives, limitations, multiplicity of analyses, results from similar studies, and other relevant evidence	9,10	Our results reflect similar findings previously published in animal models and adult TBI research. A study performed in adult patients found that ASC levels rise promptly following TBI, both in serum and CSF samples, compared to no-TBI controls 20. Additionally, our findings showing elevated caspase-1 levels at baseline in combined GCS severities, as well as in the standalone severe GCS category, are consistent with adult TBI research that associates higher levels of caspase-1 in CSF samples with clinical indicators of brain injury severity in the ICU.<br><br>An interesting finding in our results is that in mild pTBI cases from our cohort, ASC levels increased in early baseline samples in the mild GCS category, albeit with moderate sensitivity. This is especially important as the diagnosis and assessment of mild pTBI are usually challenging tasks. Existing guidelines suggest that a diagnostic approach should consist of a combination of clinical evaluation, symptom assessment, and selective use of imaging and cognitive testing tools.
Generalisability	21	Discuss the generalisability (external validity) of the study results	11	This study contributes to understanding the role of NLRP3 inflammasome activation in pediatric traumatic brain injury (pTBI) by profiling serum levels of these proteins in the acute phase of TBI and their clinical correlation with injury severity. Future research should explore the relationship between serum inflammasome protein levels and specific neuronal injury biomarkers, along with established brain injury severity prediction tools, to develop accurate, non-invasive models for predicting neurological outcomes post-TBI, especially in mild cases where severity assessment is challenging. Such models could provide valuable insights for clinicians and caregivers regarding post-TBI prognosis. Additionally, this study lays the groundwork for future investigations into the role of these proteins in therapeutic strategies for pTBI, specifically targeting inflammasomes and related proinflammatory cytokines.
**Other information**				
Funding	22	Give the source of funding and the role of the funders for the present study and, if applicable, for the original study on which the present article is based	12	The project described was supported by Grant Number UL1TR002736, Miami Clinical and Translational Science Institute, from the National Center for Advancing Translational Sciences and the National Institute on Minority Health and Health Disparities and by an R01 grant from the NIH/NINDS to R.W. Keane and J.P. de Rivero Vaccari (R01NS113969-01) and an RF1 grant from the NIH/NINDS/NIA (1RF1NS125578-01) to W.D. Dietrich and J.P. de Rivero Vaccari. Its contents

	are solely the responsibility of the authors and do not necessarily represent the official views of the NIH.<br><br>Drs Munoz Pareja and de Rivero Vaccari had full access to all the data in the study and take responsibility for the integrity of the data and the accuracy of the data analysis. Concept and design: Munoz Pareja, de Rivero Vaccari, Dhanashree, Wang. Acquisition, analysis, or interpretation of data: Munoz Pareja, de Rivero Vaccari, Mateo Chavez, Bernal, Swaby, Pringle, Guthrie, Coto, Dhanashree, Joslyn Gober, Perez, Solano, McCrea, Thorson, Kauffman, Alkhachroum, O'Phelan, Kobeissy, Wang. Drafting of the manuscript: Munoz Pareja, de Rivero Vaccari, Mateo Chavez, Bernal, Wang Critical revision of the manuscript for important intellectual content: Munoz Pareja, de Rivero Vaccari, Mateo Chavez, Bernal, Swaby, Pringle, Keane, Wang, Dietrich Statistical analysis: Munoz Pareja, de Rivero Vaccari, Mateo Chavez. Obtained funding: Munoz Pareja, de Rivero Vaccari, Wang, Dietrich. Administrative, technical, or material support: Supervision: Munoz Pareja, de Rivero Vaccari, Dhanashree, Wang, Dietrich.

*Give information separately for cases and controls in case-control studies and, if applicable, for exposed and unexposed groups in cohort and cross-sectional studies.

Note: An Explanation and Elaboration article discusses each checklist item and gives methodological background and published examples of transparent reporting. The STROBE checklist is best used in conjunction with this article (freely available on the Web sites of PLoS Medicine at http://www.plosmedicine.org/, Annals of Internal Medicine at http://www.annals.org/, and Epidemiology at http://www.epidem.com/). Information on the STROBE Initiative is available at www.strobe-statement.org.

Supplemental Figure S1. Results of Wilcoxon signed-rank tests (matched-pair analyses) evaluating within-subject changes in biomarker levels over time. Panel A: Apoptosis-associated speck-like protein containing a caspase recruitment domain (ASC). Panel B: Caspase-1.

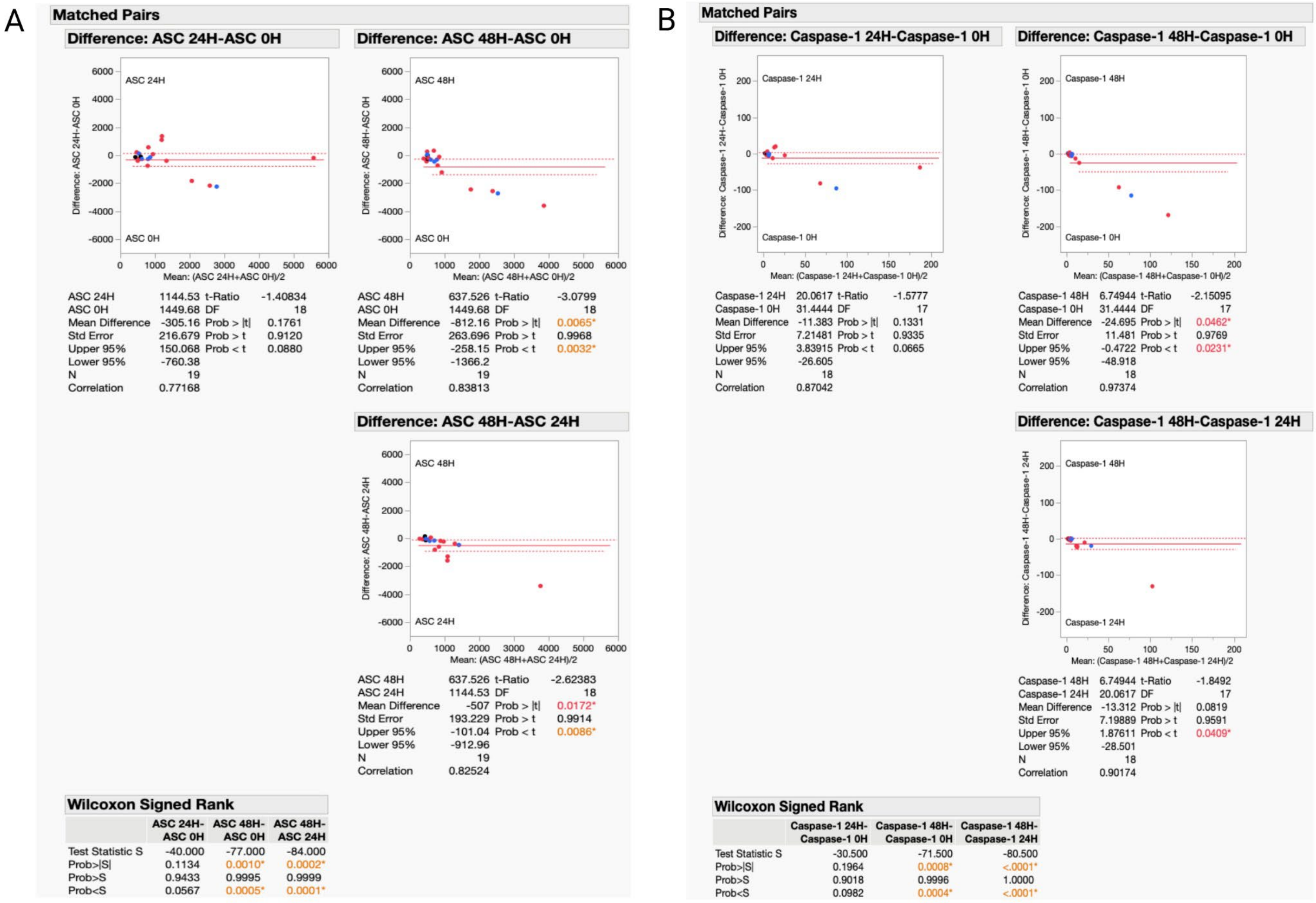

Supplement: Supplementary file 1 — Revised-Supplemental_Materials [file 41390_2025_4410_MOESM1_ESM.pdf]
